# Violations of local stochastic independence exaggerate scalability in Mokken scaling analysis of the Chinese Mandarin SF-36

**DOI:** 10.1186/s12955-014-0149-5

**Published:** 2014-10-29

**Authors:** Roger Watson, Wenru Wang, David R Thompson

**Affiliations:** Faculty of Health and Social Care, University of Hull, Hull, HU6 7RX UK; Alice Lee Centre for Nursing Studies, National University of Singapore, Singapore, Singapore; Centre for the Heart and Mind, Australian Catholic University, Melbourne, VIC 3000 Australia; Department of Psychiatry, University of Melbourne, Melbourne, VIC 3050 Australia; Department of Epidemiology and Preventive Medicine, Monash University, Melbourne, VIC 3800 Australia

**Keywords:** Mokken scaling, Stochastic independence, SF-36, Chinese Mandarin version

## Abstract

**Background:**

Previous work using Mokken scaling analysis with the SF-36 has found subscales appearing to show excellent Mokken scaling properties. However, the values of scalability of the subscales are very large, raising the possibility that these are artificially high and this may result from violations of local stochastic independence between items.

**Objectives:**

To analyse selected items from the Chinese Mandarin form of the SF-36 scale using Mokken scaling and to investigate if violations of local stochastic independence exaggerate scalability.

**Methods:**

Exploratory Mokken scaling analysis was run using the online public domain software R by entering 19 items from the Chinese Mandarin form of the SF-36 items into the analysis. The items in the resulting scales, judged by the size of Loevinger’s coefficient, were analysed for violations of monotony, 95% confidence intervals and invariant item ordering, including inspection of item pair plots.

**Results:**

Two Mokken scales were obtained, one including items from the Physical Functioning subscale, and one including items from the Mental Health subscale of the Chinese Mandarin form of the SF-36. The Physical Functioning scale was very strong according to Loevinger’s coefficient with high invariant item ordering; the Mental Health scale was moderately strong with weak invariant item ordering.

**Conclusion:**

The strength of the Physical Functioning Mokken scale derived from the Chinese Mandarin form of the SF-36 is probably the result of an item chain and item overlap which violate local stochastic independence. This is due to the nature of the items in the Physical Functioning subscale, all of which relate to physical ability and some of which can only be achieved if previous items in the subscale have been achieved.

## Background

### Local stochastic independence

Questionnaires are commonly used to measure quality of life and a prime example is the Short Form 36-item health survey (SF-36). Questionnaires are comprised of a series of questions, commonly referred to as items, which measure a latent trait (eg quality of life) and in larger questionnaires it is common for sets of items to be grouped under themes or subscales which purport to measure different aspects of the latent trait (eg physical health and mental health). The relationship between items may be merely conceptual but this is commonly supported mathematically using any one of a range of methods under the umbrella of multivariate statistics. These methods, essentially, study the way sets of items correlate or covary. However, some assumptions about the relationship between items should be met and one of these is local stochastic independence (LSI).

In the study of latent variables, LSI is a crucial property [1], whether these are being studied using methods under the umbrella of classical test theory (CTT), for example factor analysis, or item response theory (IRT), for example Rasch analsyis. When LSI can be assumed, any observed relationships between items in a questionnaire—covariance—can be assumed to be a result of the latent trait being measured rather than some other property of the items such as overlap between items or formation of items chains among items. However, LSI is usually assumed rather than estimated and, in fact, for some it is a hard concept to grasp: both its nature and its necessity.

Confusion over the nature of LSI arises because items forming scales have to covary [2] and this can imply some stochastic dependence; in other words, that variance in one item depends on the variance in another. However, the assumption of LSI implies that the item covariance is independent and, specifically, means that the observed covariance of items in a scale is a result only of the latent trait that they measure [3]. Therefore, the endorsement of any specific item is independent of the responses to other items on the latent trait. For example, if the underlying latent trait is ‘tendency to become depressed’, the items ‘I don’t feel like getting out of bed in the morning’ and ‘I feel like a worthless person’ may both be incorporated into a scale; however, the response to one item is not dependent on the other and, theoretically, both items could be responded to by anyone without endorsing the other. In reality, when they are included in a series of questions about depression, both are likely to be endorsed. In IRT, where an order in the way items are endorsed is assumed [4], the ordering of the items is also considered to be stochastically independent. The order of responses to items is a measure of the latent trait but it is not necessary to respond to one item before or after another; if items are ordered then it is assumed to be a result of the latent trait [1]. We will expand on this below under the description of Mokken scaling.

The above explains the nature of LSI. The importance of the concept arises from the fact that, without assuming LSI, it is not possible to assume anything about a putative latent trait, only about some other relationship—not necessarily a result of the latent trait—between a set of items. Item dependence arises, as explained by Bakazs and de Boeck [5] from two main sources: item chains; and item overlap. When items are in a chain, the success of any item may depend on a previous item; when items overlap, they include very similar concepts.

#### Item chains

An item chain may arise, for example, in tests of calculation where it is essential to compute the answer to one question and use that value in another question and, conversely, impossible to answer that question without answering the previous one. In the measurement of physical health, for example, an item chain would arise if a set of items were logically connected in terms of incremental ability such as ability to climb a set of stairs. If the questions were provided in numbers of steps (ie ‘Can you climb 5 steps?’; ‘Can you climb 10 steps?’) it has to be the case that someone unable to climb 5 steps will definitely be unable to climb 10 steps and so on for any number of steps.

#### Item overlap

Item overlap, for example, arises in the following questions about motivation: ‘I don’t feel like getting out of bed in the morning’ and ‘I just want to stay in bed in the morning’. In this example, it is almost inconceivable that a person would answer one positively and the other negatively. In questions about preference, a series of questions related to liking for sport such as: ‘Do you like playing football?; Do you like watching football on TV?; and Do you like going to football matches? would show considerable overlap. Such items are referred to in CTT as ‘bloated specifics’ [6] and the analogous phenomenon in regression analysis is known as multicollinearity.

### Estimating LSI

Traditionally, LSI between items has been estimated using marginal frequencies in 2 x 2 contingency tables. If the items are dependent then the value of Chi-square will be large and statistically significant; if not then the value of Chi-square will be small and not statistically significant [5]. This works well for dichotomous items; but for polytomous items, the degrees of freedom can be very large and some cells in the contingency table will have low values (down to and including 0 which are lower than the Yate’s correction can account for). For parametric IRT (Rasch models) some sophisticated methods have been reported [5] but for non-parametric IRT—Mokken scaling, which is the focus of this paper—methods remain in development [7] and are not yet available.

### Mokken scaling and LSI

Mokken scaling is a non-parametric form of IRT derived from Guttman scaling [1]. It is non-parametric in the sense that no assumption is made about the shape of the relationship between the score on an item and the score on the latent trait—the item response functions (IRF)—other than that IRFs are monotone and non-intersecting [1]. Monotony refers to a property of an IRF whereby it is continually increasing over the range of the latent trait to which it relates. Non-intersection is now more commonly referred to as invariant item ordering (IIO) [8] and is a property of IRFs whereby the IRFs for the total scores on a set of items are non-intersecting and the item step response functions (ISRFs) for each of the steps between response categories in polytomous items are also non-intersecting. When items are dichotomous, the IRFs and ISRFs are equivalent, and when they are non-intersecting IIO (formerly referred to as double monotony) also holds. Items which violate monotony can be identified in the diagnostics generated by Mokken scaling software and estimating IIO will be considered below.

Mokken scaling is described as a stochastic version of Guttman scaling because it envisages a stochastic—rather than a deterministic—relationship between the score on an item and the score on the latent trait. Nevertheless, the strength of a Mokken scale is judged by the number of Guttman errors [9], whereby the relative endorsement of pairs of items is not in the expected direction. Like parametric forms of IRT, Mokken scales—which are assumed to be unidimensional [1]—select items that form hierarchies on the basis of item difficulty. In this sense, ‘difficulty’ refers to the likelihood of an item being endorsed; where endorsement of an item is indicated by a higher score on that item, then the most difficult items will have lower mean item scores [8]. For example, in a scale which was designed to measure tendency to become depressed, an item about general lack of motivation would most likely be endorsed more readily than an item indicating suicidal ideation. Normally, we would expect the latter item to score lower than the former, and in a perfect Mokken scale (or a perfect Guttman scale) that would always be the case. However, in some cases items will be scored counter to expectations and these will be Guttman errors. The fewer the Guttman errors, the stronger the scale [3]. The extent of Guttman errors in a Mokken scale is measured using Loevinger’s coefficient H [3], a measure of scalability, which can be reported for items (Hi), items pairs (Hij) and the overall scale (Hs). For items to be retained in a Mokken scale they must have Hi higher than some predetermined lowerbound level (c) which is normally set at 0.30. Items may also be judged by the 95% confidence intervals (CIs) around Hi and the CIs should not include the lowerbound value [10]. For item pairs, the 95% CIs should not include 0 [10]. For scales, the values of Hs can be considered as follows [1]:

- Hs > 0.3 indicates a weak scale
- Hs > 0.4 indicates a moderate scale
- Hs > 0.5 indicates a strong scale.

It should be noted that strong scales and, especially, values of Hs greatly exceeding 0.50 are very rare in Mokken scales and very high values of H should be treated with caution and may indicate violations of LSI [11]. In IRT this will arise, as introduced above, if items in a scale form a chain where responding to any question is dependent on or impossible without responding to another question in the scale. An example from soccer, used in a previous paper [12], can illustrate this. If we consider that there is a latent trait ‘ability at soccer’ then, over a football season, the team that wins the league—where all other teams are played at least once—can be considered to have achieved the highest level of difficulty. A position in a league, therefore, is analogous to a scale with LSI: the winning team, for example, does not have to win every match and it does not have to win any particular matches, only to win most and gain the highest number of points. Contrast this with a soccer cup competition where each stage is a ‘knock-out’ for each team: only the winning team progresses to the next stage. Despite the fact that the latent trait of ‘ability at soccer’ may well contribute to a team’s position in the competition, a team’s position is absolutely dependent on winning the previous stage of the competition; relative position in such a competition is not, therefore, independent of performance at another level.

Invariant item ordering is estimated using a coefficient which is analogous to Loevinger’s coefficient H called Htrans (H^T^) which is a measure of how close IRFs are [13]; the closer they are then the more likely intersection is and the less likely that IIO holds; the range of values of H^T^ is as follows [13]:

- H^T^ > 0.3 indicates weak IIO
- H^T^ > 0.4 indicates moderate IIO
- H^T^ > 0.5 indicates strong IIO.

Finally, the reliability of a Mokken scale can be estimated by a reliability coefficient Rho [14], values of which should exceed 0.70 and the probability of obtaining a Mokken scale can be estimated using a Bonferroni method that accounts for multiple iterations in the method [3]; the default setting is normally p <0.05.

#### Exploratory versus confirmatory Mokken scaling analysis

Mokken scaling analysis can be applied in either an exploratory or a confirmatory mode where the same criteria are used in both modes; the only difference is what is entered into the analysis. In exploratory Mokken scaling analysis a large pool of variables about which nothing is assumed or known in relation to the existence of Mokken scales is entered into the analysis. In exploratory Mokken scaling analysis, known or assumed scales are entered into the analysis and tested against the minimum criteria for Mokken scales. The two approaches are entirely complementary and flexible in the sense that in exploring the structures of established scales, new insights into existing scales can be gained and new scales developed; there is no hierarchy of methods. In the present study, as explained below, exploratory Mokken scaling analysis was considered appropriate.

### The SF-36

The SF-36 is a generic instrument consisting of 36 questions to measure functional health and well-being from the patient’s perspective. It is a practical, reliable and valid measure of physical and mental health that can be completed in five to ten minutes. The SF-36 provides scores for eight health domains (Physical Functioning, Role-Physical, Bodily Pain, General Health, Vitality, Social Functioning), and two measures of Mental Health, and provides psychometrically-based physical component summary and mental component summary scores [15]. All items are rated on a three to six-point Likert scale, except for seven items in the role-physical and role emotional sub-scales, which are answered in a ‘yes/no’ format. The SF-36 is designed for adults 18 years of age or older and can be self-administered or interview-administered. Scores are calibrated so that 50 is the average score or norm. Because the SF-36 uses norm-based scoring, comparisons can be made among other generic health surveys (SF-12 and SF-8). The SF-36 is a robust, widely used measure of quality of life, and has been translated into different languages, including Chinese Mandarin [16]. The Chinese Mandarin version of the SF-36 (CM: SF-36) has been demonstrated to have good validity and reliability [16,17], and has been increasingly used to measure the quality of life of Chinese speaking patients, including patients with coronary heart disease [18,19].

### Mokken scaling of the SF-36

There have been two previous studies of the SF-36 using Mokken scaling [20,21]. The first study [20] was of a Dutch language version of the SF-36 and Mokken scaling was used due to its less stringent nature compared with parametric forms of IRT; as such it was considered suitable for QoL measurement. This study [20] was mainly concerned with the concepts of unidimensionality and reliability of the sub-scales of the SF-36 and Mokken scaling was considered a means of establishing unidimensionality while Cronbach’s alpha—the limitations of which are considered in the paper [20]—was used to estimate reliability. SF-36 subscales were analysed individually in a confirmatory manner; all subscales appeared to be unidimensional (Hs range 0.46 (Vitality; 4 items) – 0.84 (Bodily Pain; 2 items)) with acceptable to high Cronbach’s alpha (0.77 (General Health; 5 items) – 0.93 (Physical Functioning; 10 items)). Item H values ranged from 0.40 – 0.84, monotonicity of items was assumed and items were checked for violations of double monotonicity and it was suggested that the removal of several items with high violations of double monotonicity would lead to no violations. It should be noted that these two studies were carried out before the concept of IIO for Mokken scales had been reported and the means to calculate IIO were not available for polytomous items.

The second study [21] reported the Mokken scaling of the SF-36 in older people who participated in three cohorts of a longitudinal study of ageing. Mokken scaling was applied in a confirmatory manner to each subscale of the SF-36 and to each of the cohorts separately. Scale H was taken as a measure of unidimensionality and reliability was assessed using Cronbach’s alpha. All subscales appeared to be unidimensional (Hs range 0.42 (General Health; 5 items) – 0.83 (Physical Functioning; 10 items)) with acceptable to high Cronbach’s alpha (0.71 (Social Functioning; 2 items) – 0.92 (Physical Functioning; 10 items)). Item H values ranged from 0.38 – 0.79 but no further checks of Mokken scaling parameters were done.

The above studies have several common features. Both used MSA in a confirmatory as opposed to an exploratory manner [22], thereby assuming that the underlying dimensions of the SF-36 were robust. In fact, it may have been more appropriate in this instance to explore the structure of the SF-36 to establish how many scales were present and to investigate whether there was some other structure of subscales according to the criteria of MSA. It would also have been appropriate to consider if all of the items were suitable or present in sufficient numbers for MSA. Neither study inspected the mean item values, nor any additional MSA diagnostics, to establish if there was a sensible hierarchy of items in the subscales and neither study considered the possibility, despite the nature of some of the items in the SF-36 (to be considered below), of their being violations of LSI. Both studies used Cronbach’s alpha, as opposed to the unbiased estimator of reliability Rho [14] available with Mokken scaling packages. It is well known that, in addition to other limitations [23], Cronbach’s alpha is sensitive to the number of items in a scale [24]; specifically, alpha is inflated as the number of items increases [25] as demonstrated through Monte Carlo simulation [24], and this phenomenon is apparent in the reliability data from both studies. Furthermore, on inspection of the items of the SF-36, we consider that it is not appropriate to subject them all to MSA. For example, the response formats of the first two general health questions are not congruent and clearly overlapping. Questions 4 and 5 relating to physical and mental health have two questions in common and a third very similar question. The questions on pain (7 and 8) are not suitable for MSA as there are only two of them; there is only one question related to social health (10) and the three statements under question 11 on general health are clearly overlapping. Only the items in questions 3 and 9 measuring Physical Functioning and Mental Health, respectively, provide a sufficient number of questions for Mokken scaling. We consider some of these aspects of the above studies to be problematic and that the application of MSA to the SF-36 without fully considering the nature of Mokken scaling and the possibility that some aspects may have been violated, to be an incomplete application of the method.

#### Likelihood of violations of LSI in the SF-36

Inspecting the items of the SF-36, especially those in the measuring the physical dimensions of quality of life, it is likely that violations of LSI will take place. This is especially the case for those aspects that ask about cumulative walking distances; it is logical that ability to walk any particular distance will be predicated on walking shorter distances but may not imply ability to walk a longer distance. It is likely that violations of LSI did not take place among the items related to Mental Health.

### The present study

We suspect that the extraordinarily high values of Hs obtained in both the above studies of the SF-36 indicate that the apparent scalability is artificially high. One explanation for this is that there are violations of LSI in the subscales of the SF-36 which is exaggerating the scalability.

The present study uses exploratory MSA to analyse two dimensions of items from the Chinese Mandarin form of the SF-36 (CM: SF-36) together to determine if there are underlying dimensions according to the criteria of Mokken scaling and, subsequently, to study the nature of any scales obtained. Therefore, the research question guiding the study was: ‘Do violations of local stochastic independence exaggerate scalability in Mokken scaling analysis in the subscales of the CH: SF-36?’.

## Methods

### Participants

This is a secondary analysis of data from a cross-sectional study conducted at two university teaching hospitals in the People’s Republic of China. A convenience sample consisted of patients who had a clinical diagnosis of coronary heart disease, were older than 18 years of age, were able to comprehend Chinese and did not have a known history of psychiatric disorders or a severe co-morbidity.

Ethical approval was obtained from both teaching hospitals of Xi’an Jiaotong University. A research assistant administered the CM: SF-36 and collected demographic data from patients who agreed to participate in the study. Of 248 patients invited to participate, 202 agreed and completed the questionnaire. The mean age of these participants was 62.8 (SD =11.6) years and two-thirds of the participants were male.

### Analysis

As discussed above, inspection of the items of the SF-36 suggest that some are not suitable for MSA but also that the existence of any scales within the SF-36 had not been investigated fully using MSA. Therefore, in the present analysis we chose to analyze only the 19 items in questions 3 and 9 related, respectively, to the Physical Functioning and Mental Health aspects of the SF-36. The strategy was to check the number of putative Mokken scales present in the data and, if scales were identified, to analyze their Mokken scaling properties separately. Using the software *MSP5 for Windows* [26] and the method of Hempker *et al.* [27] and Meijer and Baneke [28], as applied by Nader *et al.* [2] and Shenkin et al. [29] the data were explored for multiple dimensions. Using incremental values of c, starting at a lowerbound c =0.05, these are increased in 0.05 increments. This is continued until an appropriate balance is found between the number of scales which are reliable (rho >0.7) and an absence of trivial scales with fewer than three items. *MSP5 for Windows* was used only to study the effect on increasing the lowerbound threshold as this software is very convenient for this analysis. The remaining analysis was carried out, as described below, using the public domain software R as this uniquely, permits the calculation of H^T^ and also the plotting of IRF pairs.

CM: SF-36 data were analysed using package ‘mokken’ (http://cran.r-project.org/web/packages/mokken/mokken.pdf) in *R* [30] (public domain software available at http://www.r-project.org/) and package ‘foreign’ (http://cran.r-project.org/web/packages/foreign/foreign.pdf) was used to convert SPSS© data into *R* data. Mokken scaling analysis (MSA) was run using the automated item selection procedure (aisp) in R. The aisp selects items and allocates them to scales in an hierarchical and iterative manner starting with those item pairs which scale best (ie with the highest values of Hij) and then building scales until no further items—ie those with Hi below the selected lowerbound threshold, for example, 0.30—can be incorporated into the scales. Items with Hi below the lowerbound value are excluded from the Mokken scale. The subsequent scales are then checked for items violating montonicity (check.monotonicity in R), reliability (check.reliability in R) and IIO (check.iio in R) and confidence intervals of Hi and Hij were calculated by hand. Item pairs were plotted (using plot(check.iio(FileR))) and inspected visually for overlap and also for ‘extreme’ items: those lying far from the remaining clusters of items which could also be, artificially, exaggerating IIO.

## Results

The procedure of using incremental lowerbound values of c supported this two scale structure. Between c =0.05 to c =0.30 only one reliable scale was apparent and at c =0.35 and c =0.40, two scales were apparent both with Hs >0.50. At c >0.40, a three scale structure was apparent but the third scale was trivial. The two scales at c =0.40 perfectly partitioned the items into those related to Physical Functioning and Mental Health. After separate analysis of these two sets of items the two Mokken scales formed from the data are shown in Tables 1 and 2. The scale in Table 1 contains items exclusively from the Physical Functioning dimension of the CM: SF-36 and the scale in Table 2 contains items exclusively from the Mental Health dimension of the CM: SF-36. None of the items in either scale violated monotonicity and the 95% CIs of all item pairs were acceptable. With one exception, the 95% Cis of the items were acceptable, therefore, no further items were excluded.

**Table 1:** **Mokken scale ‘Physical Functioning’ from the SF-36 (n = 202)**

**Item**	**Label**	**Mean**	**Hi (SE)**
9	Walking 150 meters	2.75	0.78 (0.040)
10	Bathing or dressing yourself	2.72	0.67 (0.056)
5	Climbing one flight of stairs	2.66	0.78 (0.039)
3	Lifting or carrying groceries	2.57	0.74 (0.037)
8	Walking 800 meters	2.56	0.76 (0.037)
6	Bending, kneeling, or stooping	2.56	0.70 (0.044)
2	Moderate activities, such as moving a table, cleaning the floor	2.39	0.75 (0.036)
7	Walking 1,600 meters	2.28	0.74 (0.034)
4	Climbing several flights of stairs	2.20	0.71 (0.042)
1	Vigorous activities, such as running, lifting heavy objects, participating in strenuous sports	1.54	0.59 (0.065)

*Hi* = item H; *Hs* = scale H =0.73(SE 0.031); Rho = 0.93; H^T^ = 0.70.

**Table 2:** **Mokken scale ‘Role-Emotional’ from the SF-36 (n = 202)**

**Item**	**Label**	**Mean**	**Hi (SE)**
3	Have you felt so down in the dumps that nothing could cheer you up?	4.79	0.44 (0.060)
2	Have you been a very nervous person?	4.56	0.38 (0.054)*
6	Have you felt downhearted and blue?	3.52	0.53 (0.045)
4	Have you felt calm and peaceful?^†^	3.97	0.41 (0.059)
8	Have you been a happy person?	3.66	0.44 (0.050)

*Hi* = item H; *Hs* = scale H =0.44 (SE 0.045); Rho = 0.77; H^T^ = 0.35; ^**†**^-reverse scored items; items with lowerbound 95% confidence interval <0.30; * 95% confidence interval included 0.30.

### Physical functioning

**Figure 1:**
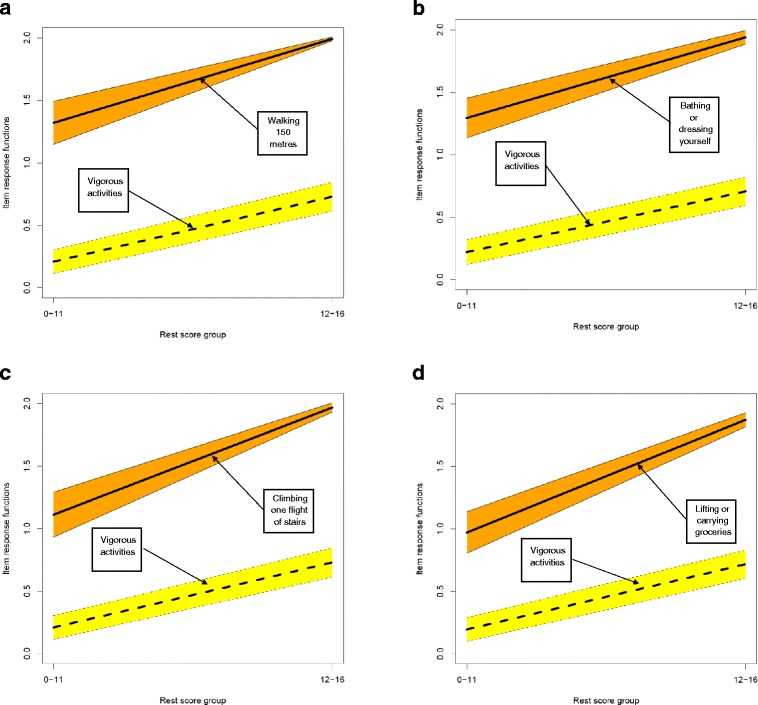
**Example of an CM: SF-36 item pair plot (for ‘Vigorous activities’) lying far from a selection of remaining item pair plots. a** Item pair plots for ‘Vigorous activities’ and ‘Walking 150 metres’. **b** Item pair plots for ‘Vigorous activities’ and ‘Bathing or dressing yourself’. **c** Item pair plots for ‘Vigorous activities’ and ‘Climbing one flight of stairs’. **d** Item pair plots for ‘Vigorous activities’ and ‘Lifting or carrying groceries’.

**Figure 2:**
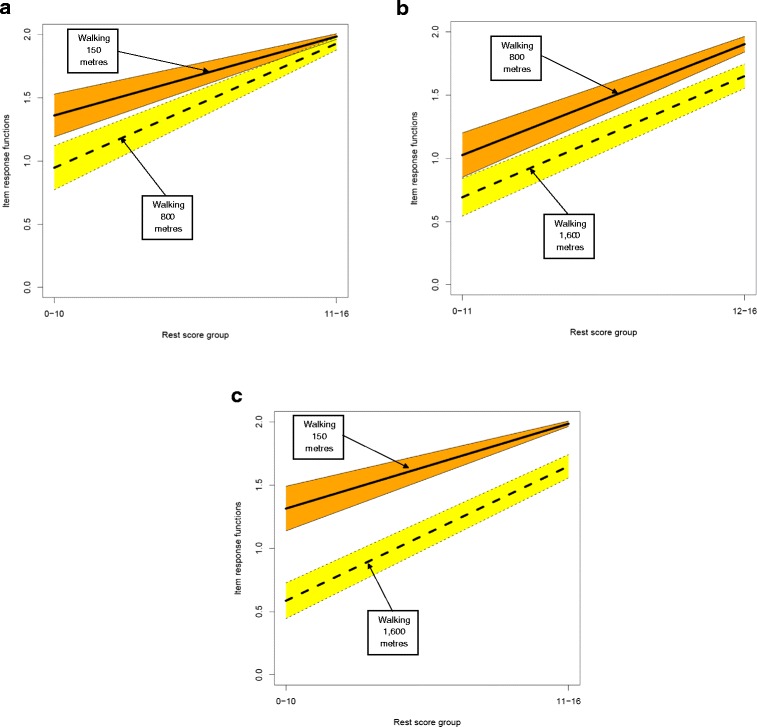
**Example of a set of items that violate local stochastic independence. a** Item pair plots for ‘Walking 150 metres’ and ‘Walking 800 metres’. **b** Item pair plots for ‘Walking 800 metres’ and ‘Walking 1,600 metres’. **c** Item pair plots for ‘Walking 150 metres’ and ‘Walking 1,600 metres’.

The Physical Functioning Mokken scale retained all 10 of the items related to that dimension in the CM: SF-36 and was a strong scale (Hs =0.73) with strong IIO (H^T^ = 0.70). Inspection of item pair plots (Figure 1) showed that the IRF for item 1 in this scale was positioned far from the remaining items and could be contributing to the high IIO; removing item 1 and re-analysis of the scale properties reduced H^T^ to 0.42 but the scale remained strong at Hs >0.70. The hierarchy of items in the Physical Functioning scale runs from walking a moderate distance through longer distances and a range of activities of daily living and instrumental activities of daily living to vigorous activity. Figure 2 shows the item pair plots for the items in the Physical Functioning scale referring to walking 150 metres, 800 metres and 1,600 metres. Collectively, the three item pair plots show increasing difficulty with increasing distance; the IRF for 800 metres lies between those for 150 metres and 1,600 metres. Items excluded from the scale were those that did not meet the minimal criteria for Mokken scaling analysis outlined in the Background section.

#### Mental health

The Mental Health Mokken scale retained all five of the items related to that dimension in the SF-36 and was moderately strong (Hs =0.44) with weak IIO (H^T^ = 0.35). However, it should be noted that, for one item the 95% confidence interval around Hi included the lowerbound 0.30 suggesting that this item could be removed from the scale. In the present study it was not removed as the confidence intervals are related to sample size and in a larger sample this item may well have 95% confidence intervals around Hi that do not include the lowerbound 0.30. It should be noted in the Mental Health scale that a low mean item score indicates endorsement and that item 4 (‘Have you felt calm and peaceful?’) is reverse scored. Therefore, from the IRT perspective, the ‘easiest’ item is ‘Have you been a happy person?’—which gained the highest endorsement—and the most ‘difficult’ item is ‘Have you felt so down in the dumps that nothing could cheer you up?’.

## Discussion

Recent work [11] has shown that high scalability in Mokken scales is worth investigating. Therefore, we set out to study if violations of local stochastic independence exaggerate scalability in Mokken scaling analysis of the subscales of the CM: SF-36 using exploratory MSA. Our view is that previous work on the SF-36 using MSA was limited. Specifically, we chose to study one subscale of the CH:SF-36 where the items were likely to violate LSI (Physical Functioning) and one where this was less likely (Mental Health). As a preliminary step, we explored the dimensionality of the Physical Functioning and Mental Health items of the CM: SF-36 using MSA to see if the underlying structure of these two subscales was supported. We consider that an exploratory approach—to see if there were subscales according to Mokken scaling criteria—was advantageous in this instance. We reiterate that some of the methods applied in this study—analysis of IIO, calculation of CIs and plotting of item pairs—were unavailable to previous analysts [20,21]. In addition, both of the previous studies of the SF-36 using MSA [20,21] used the reliability coefficient Cronbach’s alpha and not the reliability coefficient Rho which is available in Mokken scaling packages. There are well known limitations to Cronbach’s alpha [23], and Rho—described as an unbiased estimator of reliability [14]—is considered an improvement on Cronbach’s alpha and was used in this study.

The sample size in the present study was low for Mokken scaling and, until recently, little work had been done on sample size requirements. However, in simulated studies of sample size, Straat [7] has shown that one of the parameters to which sample size is related—and inversely proportional—is Hi and in this study the values if Hi, especially for the items in the Physical Functioning subscale, were high and according to Straat’s work, the sample size was probably adequate. As mentioned above, a larger sample size may have resulted in the inclusion of all the Mental Health items in the relevant Mokken scale.

Our study supports the underlying structure of the CM: SF-36 inasmuch as, according to MSA, two Mokken scales were derived in the present analysis of the 19 items related to Physical Functioning and Mental Health and each scale was derived exclusively of items related to their respective dimensions. The scale formed from the Physical Functioning subscale included ten items and had a very high Loevinger’s coefficient H. The strength of the scale is unusually high and it is apparent, on inspecting the items in the Physical Functioning subscale, there is a strong possibility they are not stochastically independent. Specifically, items 7, 8 and 9 refer to walking for increasing distances and, likewise, items 4 and 5 refer to climbing increasing numbers of flights of stairs and items 1 and 2 refer to increasing extents of exercise. These are likely to violate LSI because it is logical that achievement at any level in these incremental measures of physical activity is predicated on achievement at the lower level and that achievement above the highest level is impossible. Therefore, it is highly likely that the phenomena of an item chain is present, which is a potential sources of violations of LSI [5].

Only five items from the Mental Health subscale were retained in a Mokken scale which was moderately strong (taking the present sample size into account). It is likely that this is a true Mokken scale showing an item hierarchy that is determined by the latent trait and not due to an item chain or item overlap. Taking the fact that low scores on this subscale of the CM: SF-36 mean high endorsement, the items form a sensible hierarchy from being happy through to being impossible to cheer up and the wording of the items suggest LSI. These items are unlikely to have violated LSI because the responses are not dependent on each other and they probe different aspects of mental health. Missing items are simply a result of items failing to meet the criteria for a Mokken scale as outlined in the Methods section. Clearly this leads to some construct underrepresentation; however, in the present study this may be the result of the low sample size.

Both scales were reliable as indicated by values of Rho exceeding 0.70. For the Physical Functioning scale, Rho was very high (0.93) and this was also observed for Cronbach’s alpha in previous studies [20,21]. For the Mental Health scale, Rho was 0.77 indicating acceptable reliability. The very high levels of Rho (and Cronbach’s alpha) for the Physical Functioning scale could indicate item redundancy [31], from the scaling perspective, and this is very likely given the similar wording and overlapping concepts in the items. It should be noted that factor analysis of these items is likely to suffer from the same phenomenon, leading to an artificially highly loaded set of items on a putative factor and these items would be ‘bloated specifics’ [6]. In regression analysis these items would most likely demonstrate collinearity.

## Conclusion

Our study partly supports previous work on the SF-36 using MSA. However, we conclude that previous MSA of the SF-36 may have concluded wrongly that all the subscales were unidimensional, at least by the criteria for Mokken scales. In any case, since undimensionality in Mokken scales is related not only to simple covariance, but also to an hierarchical ordering of items in scales, MSA may not have been the most appropriate analytical procedure. This is especially the case for the items of Physical Functioning aspect of the SF-36, some of which are likely to violate LSI. We recommend in future applications of Mokken scaling, that the possibility of violations of LSI be considered either prior to the analysis and always where very high values of scalability are obtained. Nevertheless, we are aware that the properties of the CM: SF-36 may be unique and that the sample size in the present study was small.

The consequences of this study do not undermine the use of the Short Form health survey in any of its forms or translations. Indeed, further study of the English version of the SF-36—and other language translations—is warranted, especially where adequate sample sizes can be obtained. The outcome of the study does lead us to urge caution in the interpretation of putative scale properties in general—not only the SF-36—where fundamental assumptions that are crucial to the application of any psychometric method are likely to have been violated.

## Abbreviations

CM: SF-36: Chinese Mandarin SF-36
CTT: Classical test theory
H: Loevinger’s coefficient
Hi: item H
Hij: item pair H
Hs: scale H
H^T^: H trans
IIO: Invariant item ordering
IRF: Item response function
IRT: Item response theory
LSI: Local stochastic independence
MSA: Mokken scaling analysis
SF-36: Short Form 36-item health survey

## References

[1] Mokken R, Lewis JC (1982). A nonparametric approach to the analysis of dichotomous item responses. Appl Psychol Meas.

[2] Nader IW, Tran US, Baranyai P, Voracek M (2012). Investigating dimensionality of Eskin’s Attitudes Towards Suicide Scale with Mokken scaling and confirmatory factor analysis. Arch Suicide Res.

[3] Sijtsma K, Molenaar IW (2002). Introduction to nonparametric item response theory.

[4] De Ayala RJ (2009). The theory and practice of item response theory.

[5] Balazs K, de Boeck P (ᅟ). (undated) Detecting local item dependence stemming from minor dimensions.

[6] Kline P (1994). An easy guide to factor analysis.

[7] Straat JH (2012). Using scalability coefficients and conditional association to assess monotone homogeneity.

[8] Watson R, van der Ark LA, Lin L-C, Fieo R, Deary IJ, Meijer RR (2011). Item response theory: how Mokken scaling can be used in clinical practice. J Clin Nurs.

[9] Niemöller K, van Schuur W, McKay D, Schofield N, Whitely P (1983). Stochastic models for unidimensional scaling: Mokken and Rasch. Data analysis and the social sciences.

[10] Kuijpers RE, van der Ark LA, Croon MA (2013). Standard errors and confidence intervals for scalability coefficients in Mokken scale analysis using marginal models. Sociol Methodol.

[11] Egberink IJL, Meijer RR (2011). An item response theory analysis of Harter’s Self-Perception Profile for children or why strong scales should be distrusted. Assessment.

[12] Bedford A, Watson R, Lyne J, Tibbles J, Davies F, Deary IJ (2010). Mokken scaling and principal components analysis of the CORE-OM in a large clinical sample. Clin Psychol Psychother.

[13] Ligtvoet R, van der Ark LA, Marvelde JM, Sijtsma K (2010). Investigating an Invariant Item Ordering for Polytomously Scored Items. Educ Psychol Meas.

[14] Sijtsma K, Molenaar IW (1987). Reliability of test scores on nonparametric item response theory. Psychometrika.

[15] Ware JE, Kosinske M, Gandek B (2003). SF-36 Health Survey: Manual and interpretation guide.

[16] Liu CJ, Li LX, Ren XH, Li J, Zhang J, Shun D (2001). Feasibility of using Short Form 36 in Chinese population. Acad J West China Univ Med Sci.

[17] Wang W, Lopez V, Chair SY, Thompson DR (2006). The psychometric properties of the Chinese version of the SF-36 health survey in patients with myocardial infarction in mainland China. Qual Life Res.

[18] Wang W, Lau Y, Chow A, Thompson D, He H-G (2014). Health-related quality of life and social support among Chinese patients with coronary heart disease in mainland China. Eur J Cardiovasc Nurs.

[19] Wang W, Thompson DR, Ski DF, Liu M (2014). Health-related quality of life and its associated factors in Chinese patients with myocardial infarction. Eur J Prev Cardiol.

[20] Van der Heijden PMG, van Buuren S, Fekkes M, Radder J, Verrips E (2003). Unidimensionality and reliability under Mokken scaling of the Dutch language version of the SF-36. Qual Life Res.

[21] Mishra GD, Gale CR, Sayre AA, Cooper C, Dennison EM, Whalley LJ, Craig L, Kuh D, Deary IJ (2011). The HALCyon Study Team: how useful are the SF-36 sub-scales in older people? Mokken scaling of data from the HALCyon programme. Qual Life Res.

[22] Emons WHM, Sijtsma K, Pedersen SS (2012). Dimensionality of the Hospital Anxiety and Depression Scale (HADS) in cardiac patients: comparison of Mokken scale analysis and factor analysis. Assessment.

[23] Sijtsma K (2009). On the use, the misuse, and the very limited usefulness of Cronbach’s alpha. Psychometrika.

[24] DeSante CD (2011). Revisiting Reliability: The Misuse of Cronbach’s α in Political Science.

[25] Starkweather J (2012). Step out of the past: stop using coefficient alpha; there are better ways to calculate reliability.

[26] Molenaar IW, Sijtsma K, Boer P (2000). MSP5 for Windows: a program for Mokken scale analysis for polytomous items.

[27] Hempker BT, Sijtsma K, Molenaar IW (1995). Selection of unidimensional scales from a multidimensional item bank in the polytomous Mokken IRT model. Appl Psychol Meas.

[28] Meijer RR, Baneke JJ (2001). Analyzing psychopathology items: a cae for nonparametric item response theory modelling. Psychol Methods.

[29] Shenkin SD, Watson R, Laidlaw K, Starr JM, Deary IJ (2014). The attitudes to ageing questionnaire: Mokken Scaling Analysis. PLoS One.

[30] Van der Ark LA (2007). Mokken scale analysis in R. J Stat Softw.

[31] Boyle GJ (1991). Does item homogeneity indicate internal consistency or item redundancy in psychometric scales?. Pers Individ Differences.

